# The role of miR-431-5p in regulating pulmonary surfactant expression in vitro

**DOI:** 10.1186/s11658-019-0150-4

**Published:** 2019-04-02

**Authors:** Shujun Li, Zhongyi Sun, Tao Chen, Jingjing Pan, Yanqing Shen, Xiaoqing Chen, Xiaoyu Zhou, Rui Cheng, Yang Yang

**Affiliations:** 1 Department of Pediatrics, Children’s Hospital of Anhui Medical University, Hefei, China; 2 Department of Pediatrics, The First Affliated Hospital of Nanjing Medical University, Nanjing, China; 3 Department of Cardiothoracic Surgery, The First Affliated Hospital of Anhui Medical University, Hefei, China; 4 Department of Neonates, Children’s Hospital of Nanjing Medical University, No 72, Guangzhou Road, Nanjing, 210008 China

**Keywords:** miR-431-5p, Pulmonary surfactant, TGF-β/Smads pathway, Lung development

## Abstract

**Background:**

Pulmonary surfactant is the complex mixture of lipid and protein that covers the alveolar surface. Pulmonary surfactant deficiency is one of the main causes of neonatal respiratory distress. Recent studies showed that miRNA plays an important role in lung development, but research into miR-431 regulation of pulmonary surfactant are sparse. In this study, we explored the regulatory role of miR-431-5p in the expression of pulmonary surfactant and identified its potential target gene, Smad4.

**Methods:**

The bioinformatics tool TargetScan was used to predict the targets of miR-431. The expression of miR-431-5p was achieved via transfection of miR-431-5p mimics, an miR-431-5p inhibitor and corresponding negative control. The level of miR-431-5p was determined using quantitative real-time PCR. The CCK8 assay was conducted to confirm cell growth 12 h after transfection with miR-431-5p mimics, inhibitor or NC. Smad4 and surfactant-associated proteins in A549 were analyzed using western blot and quantitative real-time PCR.

**Results:**

Smad4 was validated as a target of miR-431 in A549 cells. Overexpression of miR-431 accelerated A549 proliferation and inhibited A549 apoptosis. The mRNA and protein levels for the surfactant proteins (SP-A, SP-B, SP-C and SP-D) were found to be differentially expressed in A549 cells over- or under-expressing miR-431-5p.

**Conclusion:**

Our results show that miR-431-5p is critical for pulmonary surfactant expression and that its regulation is closely related to the TGF-β/Smad4 pathway. These results will help us to study the pathophysiological mechanism of lung developmental diseases.

## Background

Pulmonary surfactant, a membrane-based lipid–protein complex, is synthesized and secreted by the type II epithelial cells of the pulmonary alveolus (AECII). It is generally stored in specific organelles, called lamellar bodies. A variety of factors have been demonstrated to be involved in the synthesis and secretion of pulmonary surfactant, such as hormonal regulation [1], genetic regulation and autocrine/ paracrine signaling [2].

To date, four proteins involved in pulmonary surfactant complexes have been identified: surfactant proteins A, B, C and D (also referred to as SP-A, SP-B, SP-C and SP-D). They mainly serve to maintain alveolar integrity, lower the surface tension of the alveolus, and suppress the inflammatory stimulus.

Immature lung development due to a lack of pulmonary surfactant is responsible for respiratory distress syndrome (RDS), which can cause severe respiratory failure. This is a major reason for mortality and morbidity in preterm babies.

A549 is a human lung adenocarcinoma cell line that has widely been used as a mature and representative model of type II pneumocytes [3]. It shows typical morphology and ultra-structural features, such as the lamellar body, which is in charge of lung surfactant synthesis and release.

MiRNAs are a class of endogenous small non-coding RNAs with functions in many physiological and pathological mechanisms, including proliferation, differentiation, apoptosis [4], inflammation [5], lipid metabolism [6], and cancer [7]. Several studies have demonstrated that miRNAs play an important role in pulmonary surfactant synthesis and fetal lung maturation [8, 9]. For example, miR-302/367 was found to be highly expressed in the early stage of lung development. Tian et al. [10] proved that alteration in miR-302/367 expression could result in an imbalance of lung endoderm progenitor proliferation and differentiation, as well as in disruption of apical basal polarity. Marcet et al. [11] found that miR-449 regulates airway epithelial multiciliogenesis through direct repression of the Delta/Notch pathway. Our previous study reported that deletion of miR-26a led to an increase in AECII and PS synthesis in rat lungs [11]. These findings provide a strong basis for the importance of miRNAs in fetal lung development and pulmonary surfactant metabolism.

To identify more significant miRNA members that are involved in the regulation of pulmonary surfactant synthesis and metabolism, we performed miRNA profiling. MiR-431 was found to have higher expression during rat lung development [9]. It also showed differential expression in the peripheral venous blood of RDS patients [8]. The above evidence indicated that miR-431 may have an important regulatory role in lung development. However, there are few relevant studies on this miRNA. It has been revealed that dysregulated expression of miR-431 relates to the occurrence of preeclampsia [12], diffuse large B-cell lymphoma [13] and hepatocellular carcinoma [13, 14], among other conditions. However, the significance of miR-431 in lung development, including its regulation of pulmonary surfactant, has been poorly explored.

Through further bioinformatics analysis based on the TargetScan database (http://www.targetscan.org/), we found that SMAD4 (*Drosophila* mothers against decapentaplegic protein 4) is a potential target gene of miR-431. The TGF-β/Smads signal pathway, which includes SMAD4, is important for lung development. This pathway is notable for its relationship with branching morphogenesis and epithelial cell differentiation during the synthesis and maturation of surfactants [15]. Smad4 is defined as the key nuclear transcription factor (Cooperating-Smad) that interacts with receptor-regulated Smads (R-Smads) to regulate the intracellular transduction of the signal from activated receptor to nucleus, integrate the signal with other signaling pathways and induce the activation of target genes [16].

In this study, we aimed to reveal the physiological function of miR-431 in surfactant protein expression and its potential regulatory mechanism through the TGF-β/Smad4 signal pathway.

## Materials and methods

### Cell culture and cell transfection

The human cell lines A549 (a non-squamous cell lung carcinoma line) and 293 T (an embryonic kidney line) were purchased from the American Type Culture Collection (ATCC). Cells were cultured in Dulbecco’s modified Eagle’s medium (DMEM; Gibco) containing 10% fetal bovine serum (FBS; Gibco), 100 mg/ml penicillin and 100 mg/ml streptomycin at 37 °C in a humidified atmosphere of 5% on 0.1% gelatin-coated culture flasks.

Cells were transfected with miR-431-5p mimics, an miR-431-5p inhibitor and the corresponding negative control (mimics-NC, inhibitor-NC; RiboBio) using Lipofectamine 3000 (Invitrogen, Thermo Fisher Scientific). Cells were plated in 6-well plates (8 × 10^4^ cells/well) or 96-well plates (2 × 10^3^ cells/well), transfected with 50 nM miR mimics or 100 nM inhibitors, incubated for 48 or 72 h, and used for further assays or RNA and protein extraction. Briefly, two mixtures were prepared separately. Mixture A consisted of 125 μl Opti-MEM (Gibco) and 5 μl Lipofectamine 3000. Mixture B consisted of 125 μl Opti-MEM and 5 μl mimic or 10 μl inhibitor. Negative control mixtures were prepared in the same way. The two mixtures were left to stand at room temperature for 5 min, then mixed and left for a further 30 min. The complex was then added to each well of the 6-well plate. The transfection efficiencies of the mimic and mimic nc were determined after 24 h, and of the inhibitor and inhibitor nc after 48 h. The miR sequence used was: hsa-miR-431-5p (accession no.: MIMAT0001625) 5′-UGUCUUGCAGGCCGUCAUGCA-3′.

### RNA isolation and extraction

Total RNA was extracted from cells using TRIzol reagent (Invitrogen, Thermo Fisher Scientific). RNA concentrations were determined using a NanoDrop ND-1000 spectrophotometer (NanoDrop Technologies).

### Detection of miR-431-5p expression via real-time PCR

MiR-431-5p was converted into cDNA from 1 μg of total RNA isolated as described above using the PrimeScript RT Reagent Kit (TaKaRa Bio) according to the manufacturer’s instructions. Real-time PCR was performed using an Applied Biosystems 7500 Fast Real-Time PCR Cycler (Applied Biosystems, Thermo Fisher Scientific). U6 was used as an internal control. The primers for miR-431-5p and U6 were purchased from Realgene. Amplification was performed with the SYBR Premix Ex Taq (TaKaRa Bio) according to the manufacturer’s protocol. Thermocycling conditions included an initial step at 95 °C for 5 min, 40 cycles at 90 °C for 15 s and at 60 °C for 15 s, 72 °C for 1 min, and a final extension at 72 °C for 10 min. The primers were: miR-431-5p forward, 5′-ACGCGTGTCTTGCAGGCCGT-3′; miR-431-5p reverse, 5′-ATCCAGTGCAGGGTCCGAGG-3′; RT primer, 5′-GTCGTATCCAGTGCAGGGTCCGAGGTATTCGCACTGGATACGACTGCATG-3′; U6 forward, 5′-CTCGCTTCGGCAGCACA-3′; U6 reverse, 5′-AACGCTTCACGAATTTGCGT-3′.

### Real-time PCR analysis of surfactant proteins and SMAD4 transcripts

Reverse transcription of 1 μg RNA isolated from A549 cells into cDNA was performed using PrimeScript RT Master Mix (TaKaRa Bio). Quantitative PCR used a SYBR Green-based detection system. Thermocycling conditions included an initial step at 95 °C for 30 s, 40 cycles at 95 °C for 5 s and 60 °C for 34 s, and final extension at 72 °C for 10 min. GAPDH primers were used for normalization. All primers were purchased from Realgene Bio-Technologies.

### Bioinformatics analysis

The bioinformatics tools TargetScan (www.targetscan.org) was used to predict the targets of miR-431. The results indicated that miR-431 was highly conserved in evolution and that the 3′-UTR of SMAD4 binds to miR-431 with a high score, suggesting SMAD4 might be a target of miR-431.

### Dual luciferase-reporter assay

The wild type (WT) and mutated (MUT) 3′-UTR sequences of SMAD4 (RiboBio), which were predicted to interact with miR-431, were inserted into a firefly luciferase-expressing pmiR-RB-REPORT vector (RiboBio) in accordance with the manufacturer’s protocol. For the luciferase reporter assay, 293 T cells were seeded into 96-well plates (1.5 × 10^4^ cells/well). Following culture for 24 h, the cells were co-transfected with the indicated vectors (50 nM miR-431 mimics and miR-431 mimic NC) using 250 ng/well Lipofectamine 3000 (Invitrogen, Thermo Fisher Scientific). At 48 h post-transfection, the cells were lysed and luciferase activity was assayed using the Dual-Luciferase Reporter Assay system (Promega). The primer was: hsa-miR-431-5p, 5′-UGUCUUGCAGGCCGUCAUGCA-3′.

### Western blot analysis

Transfected A549 cells were harvested for protein extraction using the Whole Cell Lysis Assay (KeyGEN BioTECH). Protein concentration was determined based on bicinchoninic acid (BCA) protein quantification. Approximately 40 μg protein was separated using sodium dodecyl sulfate polyacrylamide gel electrophoresis (SDS-PAGE) and then transferred onto polyvinylidene fluoride membraneolyvinylidene fluoride membranes (Bio-Rad Laboratories). The membranes were then blocked at room temperature with 5% bovine serum albumin (BSA, Biosharp) for 2 h, then incubated at 4 °C overnight with monoclonal rabbit anti-mouse SMAD4 antibody (1:2000; lot: GR3128224; Abcam), polyclonal rabbit anti-mouse SP-A antibody (1:500; lot:352231294; Sigma), polyclonal rabbit anti-mouse SP-B antibody (1:1000, lot: QC12091–42663; Novus), monoclonal mouse anti-mouse SP-C antibody (1:1000; lot: A7D107N; Novus), monoclonal rabbit anti-mouse SP-D antibody (1:1000; lot: GR121935–10; Abcam), or monoclonal rabbit anti-mouse β-actin antibody (1:2000; lot:14; Cell Signaling). After washing three times with TBST, cells were incubated with HRP-conjugated goat anti-mouse secondary antibody (1:5000; lot:293883; Abmart) or goat anti-rabbit secondary antibody (1:5000; lot:6104050; Multi Sciences) for 2 h at room temperature. Following a further three washes with TBST, the blots were visualized using ECL Plus reagents (Multi Sciences). β-actin was used as an internal control. Protein expression levels were quantified using ImageJ 2X software (version 2.1.4.5; National Institutes of Health). Finally, the protein signal was detected with an enhanced chemiluminescence (ECL) Kit (Pierce).

### CCK-8 assay

The CCK8 assay was conducted to confirm the cell growth 12 h after transfection with miR-431-5p mimics, inhibitor or NC. In brief, transfected A549 cells were seeded into 96-well plates at a density of 2000 cells per well. Staining was with 10 μl CCK-8 solution (Beyotime) per well for 1 h at 37 °C. The optical density (OD) value in each well was measured with a Multiskan GO Model 680 Microplate Reader (Thermo Fisher Scientific) at a wavelength of 450 nm. Cells were monitored every 12 h over the following 3 days.

### Flow cytometric analysis

Apoptosis of the A549 cells was evaluated with an Annexin V-FITC/PI Apoptosis Detection Kit (Multi Sciences). Cells were transfected as described above, incubated at 37 °C and 5% CO_2_ in an incubator for 48 h, and collected. After being washed twice with PBS, they were centrifuged and re-suspended in 500 μl of binding buffer. Annexin V-FITC (Cell Cycle Staining Kit, Multi Sciences). 5 μl of PI was added and mixed gently with the cells. They were then incubated at room temperature in the dark for 15 min. The wavelength of 488 nm was activated using a BD FACSCanto II flow cytometer (BD Biosciences) to determine the apoptotic rate. The experiment was repeated three times. Data analysis was performed with FlowJo software version 7.2.

### Statistical analysis

Data are expressed as means ± SD. Student’s unpaired *t*-test was used to determine significant differences. * indicates *p* < 0.05, ** indicates *p* < 0.01, and *** indicates *p* < 0.001. *p* < 0.05 was considered to indicate a statistically significant difference. All analyses were performed using SPSS 19.0 software and EXCEL 2007.

## Results

### Transfection efficiency of the miR-431-5p mimics, inhibitor and NC

**Fig. 1:**
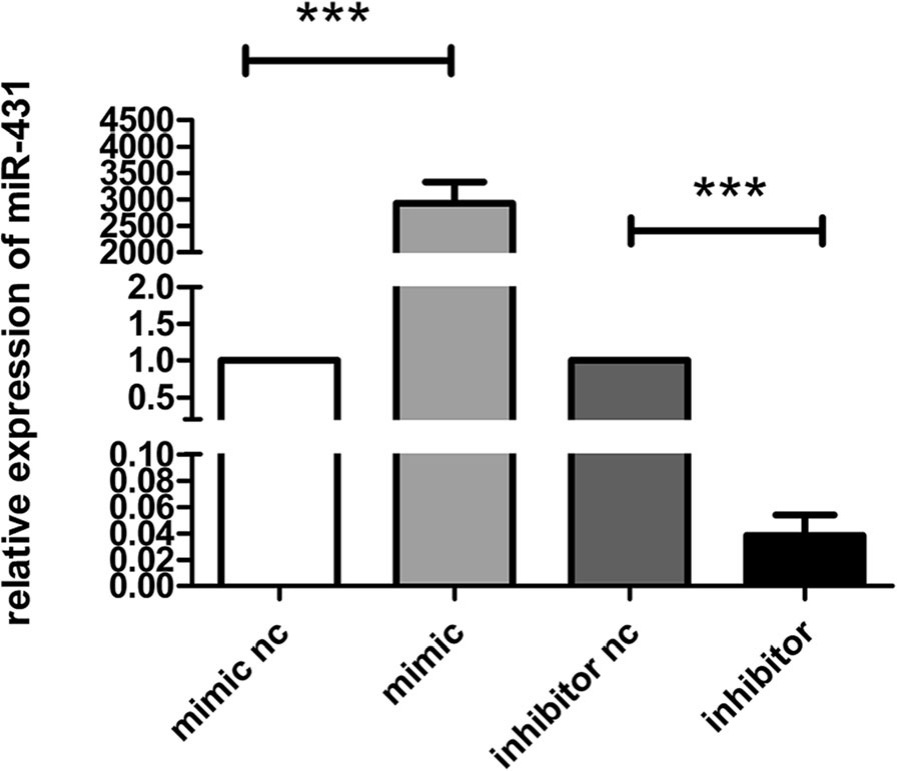
The miR-431-5p expression in A549 cells transfected with miR-431-5p-mimics and miR-431-5p-inhibitor were validated using quantitative Real-time PCR. ***p < 0.001 vs. miR-431-5p negative control

To confirm the transfection efficiency of the miR-431-5p mimics, inhibitor and NC in A549, relative miR-431-5p expression was confirmed using quantitative Real-time PCR. MiR-431-5p mimic transfection significantly increased the expression of miR-431-5p in cells compared with mimic-NC transfection (Fig. 1). MiR-431-5p inhibitor significantly decreased the expression of miR-431-5p compared with inhibitor-NC (*p* < 0.001).

### Effect of miR-431-5p on the proliferation of A549 cells

**Fig. 2:**
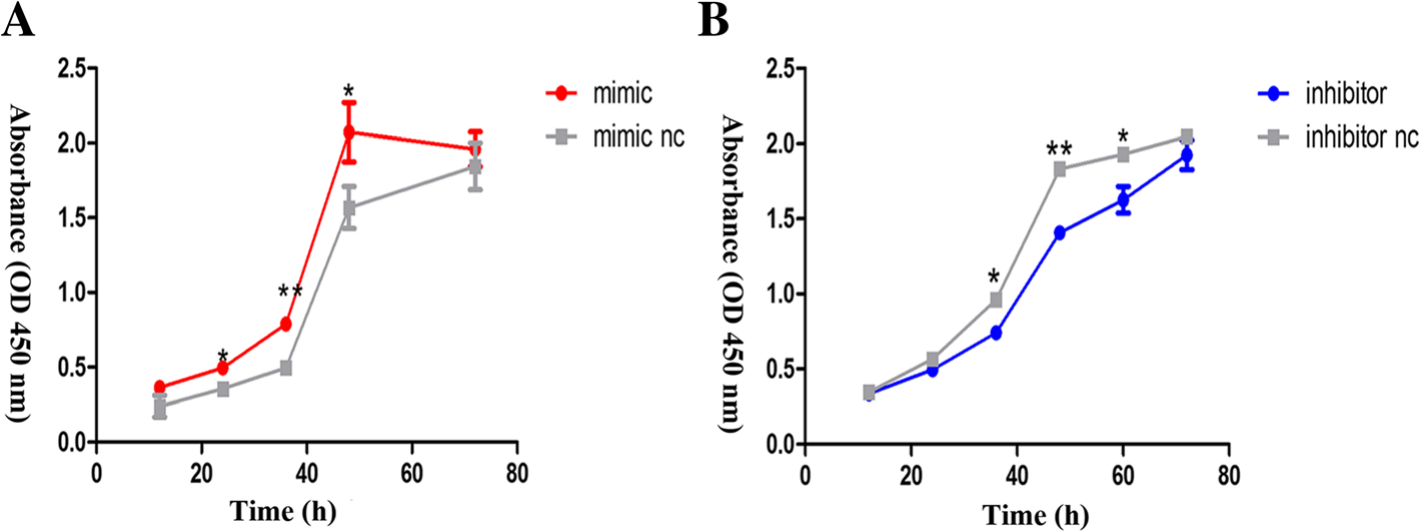
miR-431-5p promoted A549 proliferation in vitro. **a** and **b** – CCK8 assay of cells transfected with miR-431-5p mimic, inhibitor, mimic nc or inhibitor nc. *p < 0.05; **p < 0.01 vs. miR-431-5p negative control

To determine the effect of miR-431-5p on proliferation in A549 cells, we carried out the CCK-8 assay. The results showed that overexpression of miR-431-5p accelerated proliferation when compared with the control group. By contrast, downregulated miR-431-5p showed different results (Fig. 2).

### Effect of miR-431-5p on the apoptosis of A549 cells

**Fig. 3:**
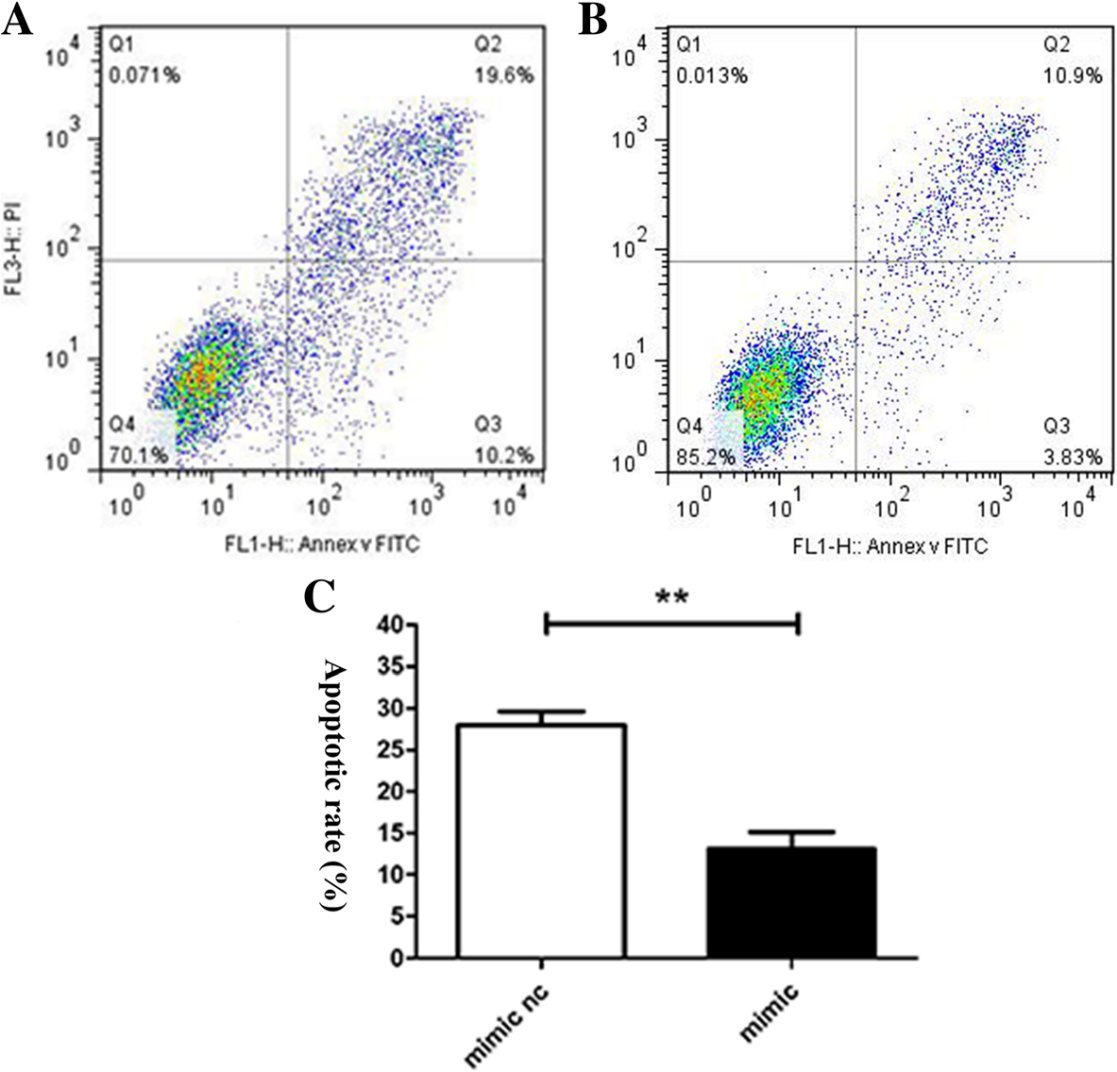
Apoptosis was assayed based on the binding of propidium iodide/AV-FITC. **a** – miR-431-5p mimic NC group. **b** – miR-431-5p mimic group. **c** – Flow cytometry was used to assess the apoptotic rate (*n* = 3). **p < 0.01 vs. miR-431-5p negative control

**Fig. 4:**
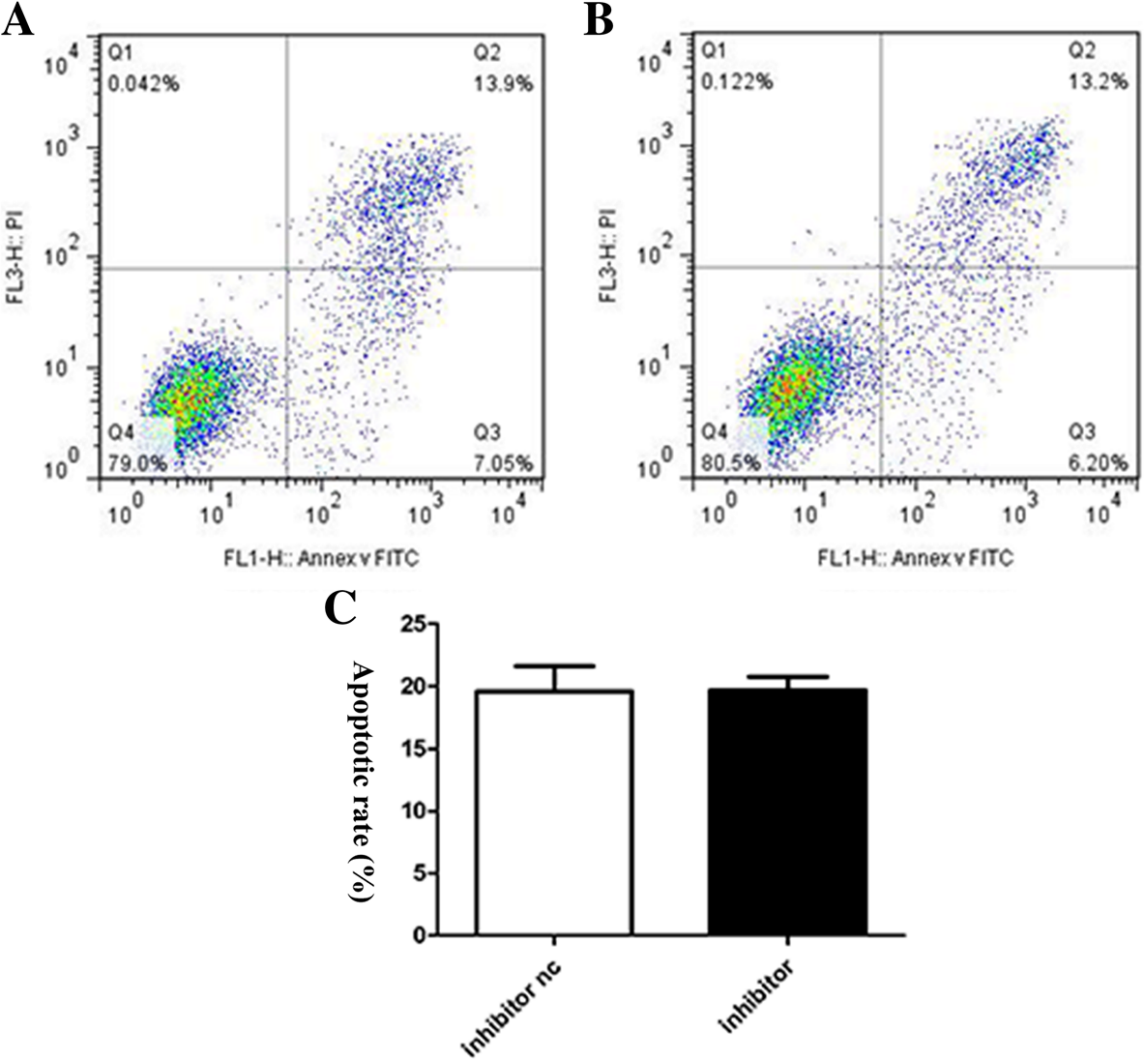
Apoptosis was assayed based on the binding of propidium iodide/AV-FITC. **a** – miR-431-5p inhibitor NC group. **b** – miR-431-5p inhibitor group. **c** – Flow cytometry was used to assess the apoptotic rate (*n* = 3) **p < 0.01 vs. miR-431-5p negative control

Compared with the miR-431 mimic NC group, the apoptosis rates in the mimic group significantly decreased (Fig. 3; *p* < 0.05). However, no significant differences in apoptosis rates were observed between the miR-431 inhibitor group and the inhibitor NC group (Fig. 4; *p* > 0.05).

### The regulatory relationship between miR-431 and SMAD4

**Fig. 5:**
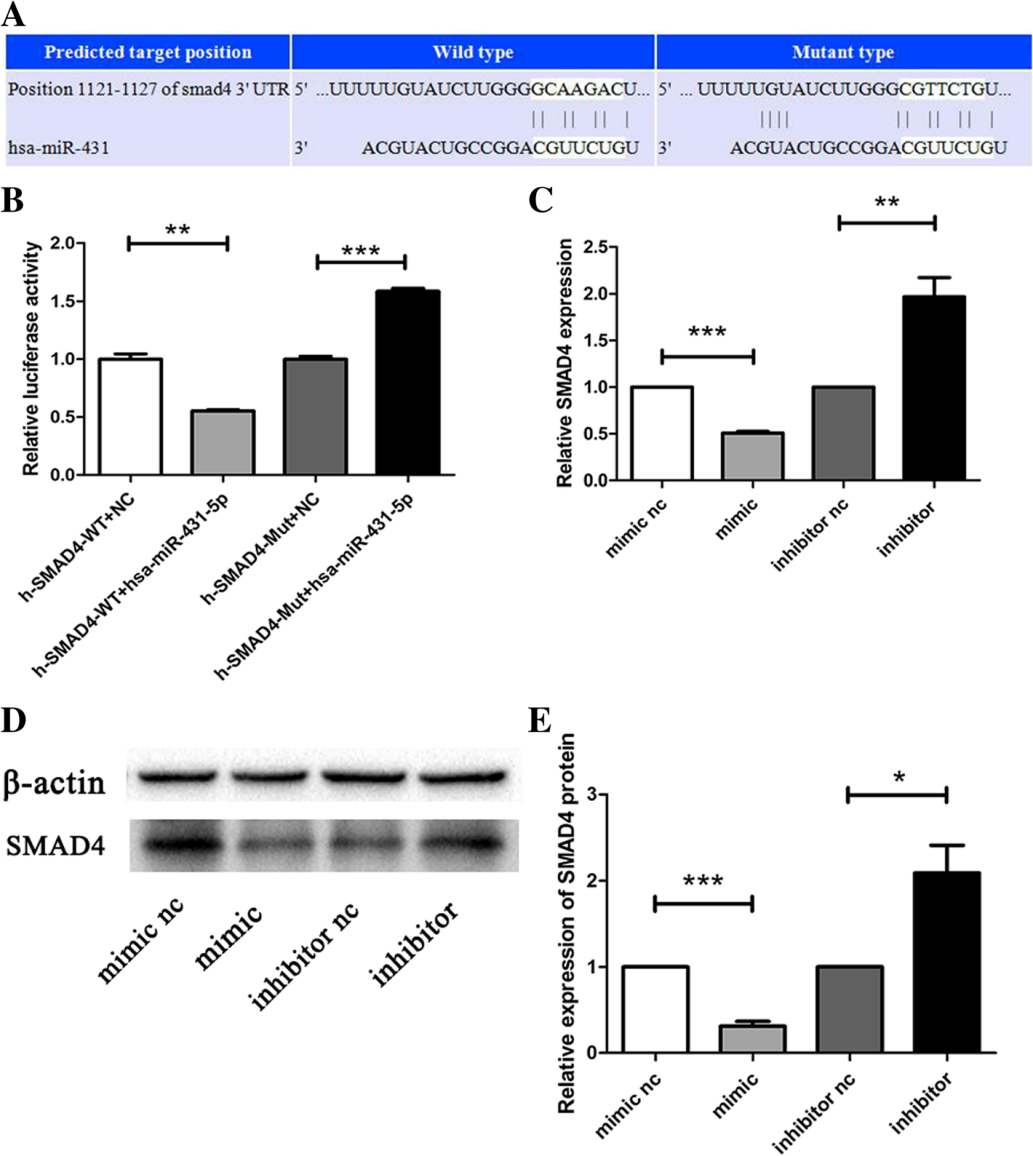
MiR-431 reduces SMAD4 expression by targeting the SMAD4 3′-UTR in A549 cells. **a** – TargetScan showing the subcloning of the predicted miR-431-binding site at position 1121–1127 of the SMAD4 3′-UTR into a luciferase construct. **b** – Luciferase reporter gene assay verification of the bioinformatics analysis. SMAD4 was predicted as a target gene of miR-431 (*n* = 3). **c** – The mRNA expression of SMAD4 in A549 cells transfected with miR-431-5p mimic, mimic nc, inhibitor or inhibitor nc. **d** – Western blotting of SMAD4 in A549 cells transfected with miR-431-5p mimic, mimic nc, inhibitor or inhibitor nc. **e** – The protein expression of SMAD4 in A549 cells transfected with miR-431-5p mimic, mimic nc, inhibitor or inhibitor nc (*n* = 3). *p < 0.05; **p < 0.01; ***p < 0.001 vs. miR-431-5p negative control

We used the TargetScan prediction database to search for downstream target(s) of miR-431. As shown in Fig. 5a, SMAD4 was predicted as one of the target genes that contained putative binding sites for miR-431.

To verify the accuracy of the prediction, we applied dual luciferase reporter assays. Upregulation of miR-431 significantly downregulated the luciferase activity of the SMAD4-WT 3′-UTR (Fig. 5b; *p* < 0.01). We found that the relevant luciferase activity in 293 T cells transfected with a mutant SMAD4 (SMAD4-mut) was elevated in the miR-431 mimic when compared with the negative control (NC) group (*p* < 0.001).

Furthermore, we detected the basic levels of SMAD4 mRNA and protein in A549. The results showed that the SMAD4 mRNA and protein levels were significantly lower in the mimic group compared with the mimic NC group. In addition, the miR-431-5p inhibitor showed the opposite result in A549 cells (Fig. 5c–e).

### Expression of surfactant proteins regulated by miR-431

**Fig. 6:**
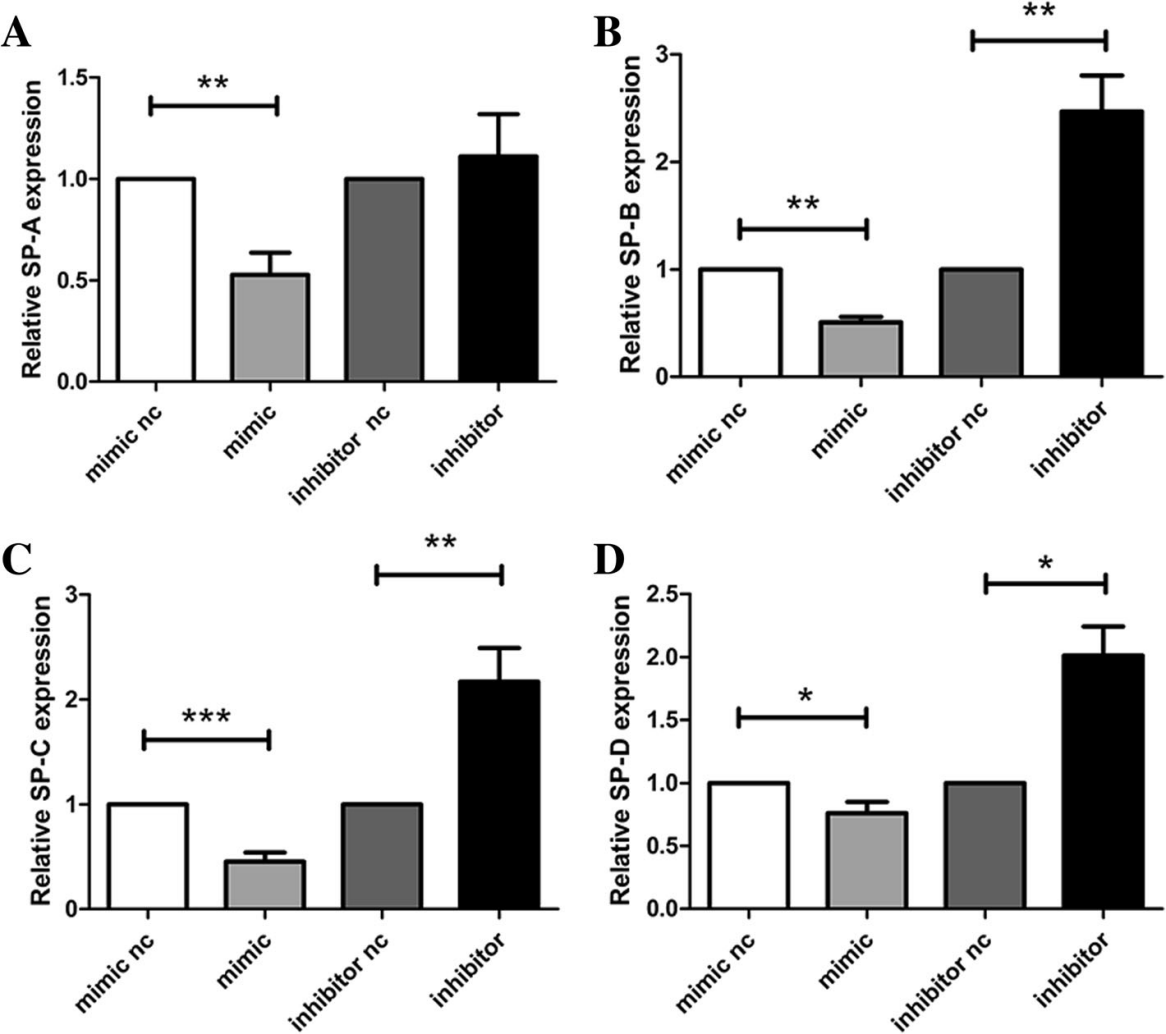
Quantitative real-time PCR was used to detect the expression of surfactant proteins. **a** – SP-A mRNA expression in A549 cells. **b** – SP-B mRNA expression in A549 cells **c** – SP-C mRNA expression in A549 cells. **d** – SP-D mRNA expression in A549 cells. *p < 0.05; **p < 0.01; ***p < 0.001 vs. miR-431-5p negative control

To determine whether pulmonary surfactant synthesis is regulated by miR-431 in A549 cells, the surfactant proteins SP-A, SP-B, SP-C and SP-D were detected in further experiments. Through quantitative real-time PCR, their mRNA levels were found to decrease in A549 cells overexpressing miR-431-5p. Downregulation of miR-431-5p has the opposite effect on pulmonary surfactant protein expression in A549 cells (Fig. 6a–d) in comparison with the negative controls (*p* < 0.05).

**Fig. 7:**
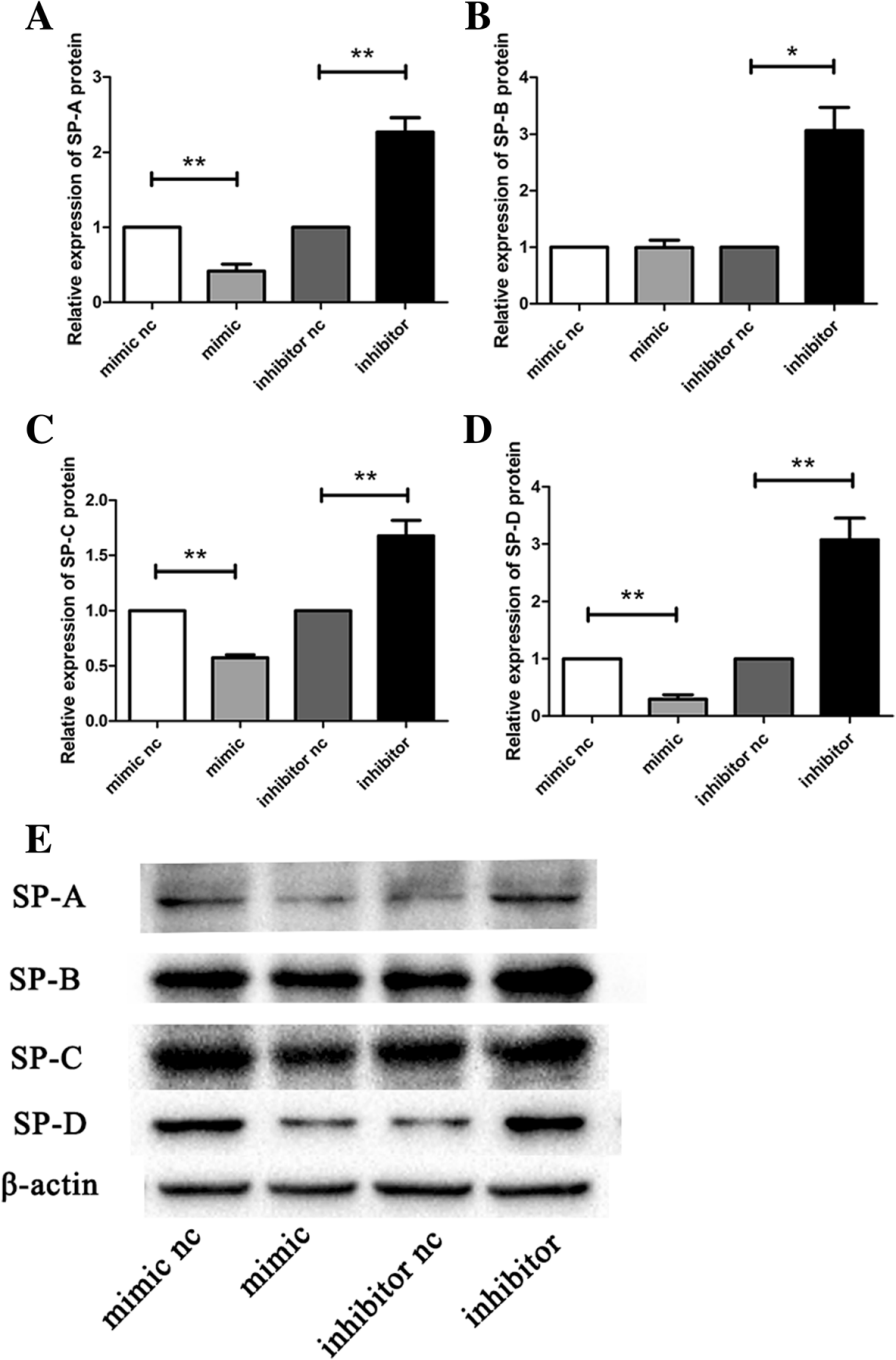
Western blotting is used to detect the protein expression of surfactant proteins. **a** – Relative expression of SP-A protein. **b** – Relative expression of SP-B protein. **c** – Relative expression of SP-C protein. **d** – Relative expression of SP-D protein. **e** – Western blotting results for, from left to right: mimic nc, mimic, inhibitor nc, inhibitor. *p < 0.05; **p < 0.01; ***p < 0.001 vs. miR-431-5p negative control

To confirm this effect at the protein level, A549 cells were harvested for western blot analysis. Compared with the negative control cells, overexpression of miR-431-5p lowered the expression of surfactant proteins. Inhibition of miR-431 promoted the expression of surfactant proteins (Fig. 7).

## Discussion

A deficiency of pulmonary surfactant due to lung immaturity leaves the organism susceptible to respiratory distress syndrome (RDS), which is one of the major causes of death in preterm infants. Pulmonary surfactant is synthesized and secreted by AECIIs and consists of lipids and proteins that cover the alveolar surfaces. The surfactant proteins include SP-A, SP-B, SP-C and SP-D. miRNAs are a class of endogenous small non-coding RNAs with functions covering almost every physiological and pathological mechanism, including proliferation, differentiation and apoptosis. TGF-β is a key regulator in lung development and diseases. When TGF-β signaling is dysregulated or essential control mechanisms are unbalanced, the resulting pulmonary system dysfunction can have profound consequences. Previous studies indicate that TGF-β1-deficient mice show systemic inflammation. AGeneralized perivasculitis or interstitial pneumonia is observed in the lungs [17]. Similarly, TGF-β2 deletion in mice results in perinatal death due to respiratory failure and structural abnormalities of the lungs [18].

In this study, which focused on the effect of miR-431 on surfactant protein expression in A549 cells, we found SMAD4 as one of the potential target genes of miR-431. However, we also found that there is a statistical difference between the Smad4-mut + miR-431-5p and Smad4-mut + NC group. One possible explanation is that microRNAs are multipotent, and the expression of the target genes can be influenced by other unknown factors.

The results of the dual luciferase reporter assay reflected the interaction of miR-431 with various molecules in cells. This result should therefore be viewed dialectically. Consistent with the above finding, Lee KP et al. [19] found that miR-431 regulates SMAD4 expression through direct binding to the Smad4 3′-UTR, and that SMAD4 protein levels were lower in old myoblasts transfected with the miR-431 mimic. Thus, the mouse miR-431 seed sequence in the Smad4 3′-UTR is conserved in different tissues. SMAD4, as the common mediator of TGF-β family with activated R-SMADs (SMAD 1, 5 and 8), is the common mediator between the TGF-β family and activated R-SMADs (SMAD 1, 5 and 8). They together form trimeric complexes which are translocated to the nucleus, where they cooperate with other transcription factors, co-activators and co-repressors to regulate the expression of specific genes.

Overexpression of miR-431 inhibited the expression of surfactant proteins. However, we have not yet investigated the direct relationship between TGF-β/SMAD4 signaling and surfactant protein expression.

Previous studies demonstrated that lung development (including maturation of alveolar epithelial type II and surfactant protein expression) is associated with TGF-β/Smad4 family. For example, TGF-β inhibits alveolar epithelial cell growth and repair [20]. Qiu L et al. [21] reported that TGF-β1 differentially downregulates the expression of three (SP-A, SP-B and SP-C) acting at the gene transcription level. Our study showed that increased expression of miR-431-5p in A549 may contribute to lower expression of SMAD4, and eventually leading to the downregulation of surfactant proteins. The relationship between TGF-β/Smad4 signaling and lung development is certainly complex.

For instance, Smad3 could generally amplify TGF-β signal, and conditional knockout of the Smad3 gene can cause alveolar expansion in diseases such as bronchopulmonary dysplasia (BPD) or emphysema. This suggests that TGF-β may play a positive role in regulating the late stage of lung development [22, 23]. Interestingly, artificial upregulation of the TGF-β signal also leads to alveolar enlargement, suggesting that the conditional overexpression of TGF-β signal may also play a positive role in the formation of BPD [24, 25].

Another example showed that overexpression of the TGF-β1 ligand in the lung interferes with certain alveolar functions during later lung development, causing states such as stagnation of epithelial cell differentiation and inhibiting pulmonary angiogenesis and final lung development [26, 27]. All this evidence proves that TGF-β has a negative regulatory effect on alveolization. However, similar physiological phenomena were found after blocking the TGF-β signaling pathway via gene knockout of Smad3 in P7 and P28 mice. This suggests that TGF-β may to some extent also play a positive role in regulating alveolization [22].

Thus, theoretically speaking, TGF-β/Smad4 signaling does not simply inhibit the expression of surfactant proteins and play a mere negative feedback role. Our study focused on the role of miR-431 in regulating surfactant protein expression. The current findings lead us to speculate that a regulating role of miR-431-5p in surfactant protein expression may be associated with activation of TGF-β/SMAD4 signaling. Further research is definitely needed to explore the relationship between Smad4/TGF-β and surfactant protein expression in lung development. In our opinion, there may be other regulatory mechanisms involved.

In this study, we found that miR-431 promoted A549 proliferation and inhibited apoptosis. We also revealed that miR-431 is a novel RDS-related miRNA that could regulate pulmonary surfactant expression. Only a few miRNAs have been identified as associated with expression of pulmonary surfactant, e.g., miR-206 [28], miR-150 [29] and miR-375 [30]. MiR-431 was previously reported to be involved in the maturation of organs, including the nervous system [31], mammary glands [32] and muscles [33]. This is the first report of a potential role of miR-431 in regulating lung development.

In conclusion, this study demonstrated that the miR-431-5p is critical for pulmonary surfactant expression. Its regulation mechanism is closely related to the TGF-β/Smad4 pathway, but further research is needed to reveal the details of this mechanism. These results will help us to study the pathophysiological mechanism of lung developmental diseases such as RDS and BPD.

## Abbreviations

BPD: bronchopulmonary dysplasia
NC: negative control
RDS: respiratory distress syndrome
